# Cancer upregulated gene 2 induces epithelial-mesenchymal transition of human lung cancer cells via TGF-β signaling

**DOI:** 10.18632/oncotarget.13867

**Published:** 2016-12-10

**Authors:** Sirichat Kaowinn, Jeonghyo Kim, Jaebeom Lee, Dong Hoon Shin, Chi-Dug Kang, Dae-Kee Kim, Soojin Lee, Min Kyung Kang, Sang Seok Koh, Seong-Jin Kim, Young-Hwa Chung

**Affiliations:** ^1^ BK21+, Department of Cogno-Mechatronics Engineering, Pusan National University, Busan 609-735, Republic of Korea; ^2^ Department of Pathology, School of Medicine, Pusan National University, Yangsan 626-870, Republic of Korea; ^3^ Department of Biochemistry, School of Medicine, Pusan National University, Yangsan 626-870, Republic of Korea; ^4^ Graduate School of Pharmaceutical Sciences, College of Pharmacy, EwhaWomans University, Seoul 120-750, Republic of Korea; ^5^ Department of Microbiology and Molecular Biology, Chungnam National University, Daejeon 305-764, Republic of Korea; ^6^ Department of Biological Sciences, Dong-A University, Busan 604-714, Republic of Korea; ^7^ CHA Cancer Institute and Department of Biomedical Science, CHA University, Seoul 135-081, Republic of Korea

**Keywords:** CUG2, TGF-β, EMT, Sp1, Smad2/3

## Abstract

Cancer upregulated gene 2 (CUG2) enhances cell migration and invasion, but the underlying mechanism has not been revealed. Herein, CUG2 decreased the expression of E-cadherin and increased the expression of N-cadherin and vimentin, characteristics of the epithelial-mesenchymal transition (EMT). A CUG2 deletion mutant, lacking interaction with nucleophosmin 1 (NPM1), or suppression of NPM1 reduced wound healing and cell invasion, indicating that CUG2-mediated EMT requires NPM1. CUG2 enhanced activation of Smad2/3 and expression of Snail and Twist, while the CUG2 silence decreased these TGF-β signaling pathways, leading to suppression of EMT. NPM silence also inhibited the CUG2-induced TGF-β signaling. These results suggest that TGF-β signaling is involved in CUG2-induced EMT. Treatment with EW-7197, a novel inhibitor of TGF-β signaling, diminished CUG2-mediated EMT and inhibition of Akt, ERK, JNK, and p38 MAPK, non-canonical TGF-β signaling molecules, also decreased expression of Smad2/3, Snail and Twist, leading to inhibition of EMT. The results confirm that TGF-β signaling is essential for CUG2-mediated EMT. Interestingly, TGF-β enhanced CUG2 expression. We further found that both CUG2-induced TGF-β production and TGF-β-induced CUG2 up-regulation required a physical interaction between Sp1 and Smad2/3 in the CUG2 and TGF-β promoter, as demonstrated by a promoter reporter assay, immunoprecipitation, and ChIP assay. These results indicated close crosstalk between CUG2 and TGF-β. Conversely, suppression of CUG2 or NPM1 did not completely inhibit TGF-β-induced EMT, indicating that the effect of TGF-β on EMT is dominant over the effect of CUG2 on EMT. Collectively, our findings suggest that CUG2 induces the EMT via TGF-β signaling.

## INTRODUCTION

Cancer upregulated gene 2 (CUG2) was identified as a candidate gene that is commonly up-regulated in various tumor tissues, such as ovarian, liver, colon, and lung, and is known to play a crucial role in tumorigenesis [1]. CUG2 was mapped to chromosome 6q22.32; it spans about 8.5 kb with a three-exon structure and encodes an 88-amino acid polypeptide [1]. We and other groups have revealed that CUG2 is a newly identified centromere component that is required for proper kinetochore function during cell division [2, 3]. CUG2 has been shown to exert an oncogenic effect in a transplant model using NIH3T3 cells expressing CUG2, in a manner similar to Ras [1]. Whereas CUG2 overexpression activates Ras and MAPKs including p38 MAPK, which eventually facilitates oncolytic reoviral replication [4], CUG2 confers resistance to oncolytic vesicular stomatitis virus infection [5] and induces faster cell migration and anti-cancer drug resistance through activation of Stat1 [6].

TGF-β is a pleiotropic cytokine that controls numerous biological functions such as proliferation, apoptosis, embryonic patterning, stem cell maintenance, cell differentiation, migration, and regulation of the immune system [7]. Regarding its effect on tumor development, TGF-β has been shown to have two actions: tumor suppression and tumor promotion that highly depend on cell type and context [8, 9]. For example, TGF-β acts as a tumor repressor during early tumor growth, resulting in growth arrest and apoptosis, whereas TGF-β also initiates cancer progression and metastasis through Smad-dependent [10] or -independent signaling pathways [11]. Smad-dependent signaling regulates the epithelial-mesenchymal transition (EMT), a prelude to cancer progression, through the expression of Snail, ZEB, and Twist [12]. These transcriptional regulators repress the expression of epithelial markers such as E-cadherin, occludin and plakoglobin but enhance mesenchymal markers including vimentin, fibronectin, and N-cadherin [12, 13]. TGF-β signaling involves ligand binding to TGF receptors (TbR1 and TbR2). Smad2/3 is subsequently activated and forms a complex with Smad4, which leads to translocation to the nucleus and interaction with other transcription factors to regulate the expression of target genes [14, 15]. The Smad-independent pathway activates PI-3 kinase/Akt, and the Ras-ERK signaling axis and also promotes JNK and p38 MAPK pathways via the TRAF6-TAK1signaling axis [16, 17].

This study was initiated to determine how CUG2 contributes to metastasis in tumor development. We herein report that CUG2 induces the EMT in human lung cancer cells via enhancement of TGF-β signaling. Of interest, TGF-β signaling reversely increases the expression of CUG2. We also found that Sp1 and Smad2/3 are involved in crosstalk between CUG2 and TGF-β. Furthermore, inhibition of TGF-β signaling blocks the CUG2-mediated EMT, indicating that TGF-β signaling is a potential target for CUG2-mediated oncogenesis.

## RESULTS

### CUG2 enhances the EMT, which requires interaction between CUG2 and NPM1

**Figure 1:**
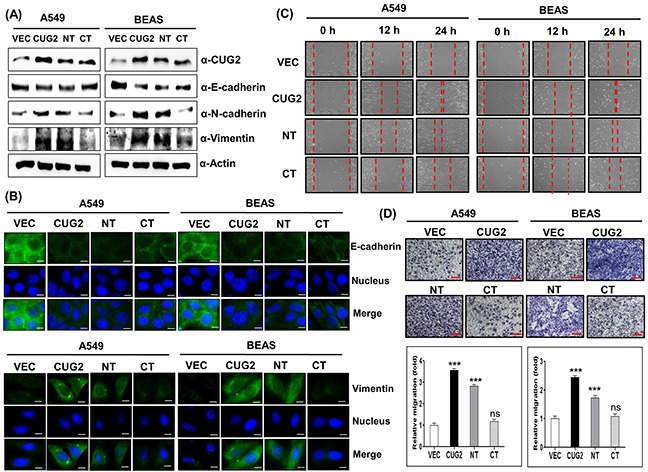
CUG2 induces EMT, in which NT of CUG2 is more important than CT of CUG2 **A**. Expression of CUG2, E-cadherin, N-cadherin, and vimentin was detected by immunoblotting using the corresponding antibodies. NT indicates N-terminal domain of CUG2 and CT indicates C-terminal domain of CUG2. **B**. Expression of E-cadherin and vimentin was detected by immunofluorescence using Alexa Fluor 488-conjugated goat anti-mouse IgG (green) and Alexa Fluor 488-conjugated donkey anti-goat IgG (green), respectively. For nuclear staining, DAPI was added prior to mounting in glycerol. Scale bar indicates 10 μm. **C**. Cell migration was measured by a wound healing assay. The wound closure areas were monitored by phase-contrast microscopy at a magnification of 100×. The assays were repeated twice. **D**. An invasion assay was performed using 48-well Boyden chambers. The chamber was assembled using polycarbonate filters coated with Matrigel. Scale bar indicates 100 μm. The assays were repeated twice. Each assay was performed in triplicate and error bars indicate standard deviation (SD) (ns; not significant, *p*> 0.05, ***; *p*< 0.001)

Since our previous studies showed that overexpression of CUG2 increases cell migration [6], we explored whether CUG2 also plays a critical role in the EMT, a key process in tumor invasion and migration. Although CUG2 was overexpressed in various tumor tissues, such as lung, ovarian, liver, and colon [1], lung cancer cell model has been introduced due to high morbidity and mortality. Because A549 cancer cells are well-known as a non-small cell lung cancer line, we used the cells in the study to address the role of CUG2. Furthermore, we used an immortalized BEAS-2B cell line derived from the bronchus to reproduce a CUG2-induced phenotype seen in A549 cell line. We first examined typical features of the EMT such as decreases in E-cadherin expression, and increases in N-cadherin and vimentin protein levels in A549 lung cancer cells (A549-CUG2) and immortalized bronchial BEAS-2B cells (BEAS-CUG2) stably expressing CUG2. We then found that A549-CUG2 and BEAS-CUG2 cells exhibited decreases in E-cadherin expression and increases in N-cadherin, and vimentin protein levels compared to those in A549-Vec and BEAS-Vec cells (Figure 1A), which were confirmed by immunofluorescence (Figure 1B). Consequently, A549-CUG2 and BEAS-CUG2 cells recovered much faster from wound healing and invaded more aggressively than A549-Vec and BEAS-Vec cells over 24 h (Figures 1C and 1D). Furthermore, we observed no difference in cell proliferation between A549-CUG2, BEAS-CUG2 and their control cells over 24 h (Supplementary Figure S1), which can exclude the possibility that faster cell growth due to CUG2 overexpression causes the enhanced wound healing and cell invasion. On the other hand, our previous study demonstrated that CUG2 interacts with NPM1, a multifunctional nuclear phosphoprotein and revealed that the N-terminal domain (amino acids 1-66) of CUG2 binds to NPM1 but the C-terminal domain (amino acids 31-88) does not [18]. We thus produced A549 and BEAS-2B cells stably expressing CUG2's N-terminal domain (A549-CUG2NT; BEAS-CUG2NT) and C-terminal domain (A549-CUG2CT; BEAS-CUG2CT). As seen in Figure 1A and 1B, A549-CUG2NT and BEAS-CUG2NT cells displayed down-regulation of E-cadherin and up-regulation of N-cadherin and vimentin, similarly to A549-CUG2 and BEAS-CUG2 cells. However, A549-CUG2CT cells showed similar levels of E-cadherin as A549-Vec cells while BEAS-CUG2CT cell displayed slightly lower levels of E-cadherin than BEAS-Vec cells. Nevertheless, E-cadherin protein levels in BEAS-CUG2CT were still higher than those in BEAS-CUG2 and BEAS-CUG2NT cells. Protein levels of N-cadherin in A549-CUG2CT and BEAS-CUG2CT cells were similar to those in A549-Vec and BEAS-Vec cells, respectively. Protein levels of vimentin in A549-CUG2CT cells were similar to those in A549-Vec cells while the vimentin protein levels in BEAS-CUG2CT cells were slightly higher than those in BEAS-Vec cells. Consequently, A549-CUG2NT and BEAS-CUG2NT cells performed slightly slower in wound-healing assays than A549-CUG2 and BEAS-CUG2 cells (Figure 1C). A549-CUG2NT and BEAS-CUG2NT cells also showed slightly slower invasiveness compared to A549-CUG2 and BEAS-CUG2 cells (Figure 1D). However, A549-CUG2CT and BEAS-CUG2CT cells exhibited similar results to A549-Vec and BEAS-Vec cells in wound healing and cell invasion assays (Figures 1C and 1D). Taken together, these results suggest that the NT domain of CUG2 seems to be more important in CUG-mediated EMT than the CT domain of CUG2. The results indicate that the NT domain of CUG2 is necessary but not sufficient for CUG2-mediated EMT.

**Figure 2:**
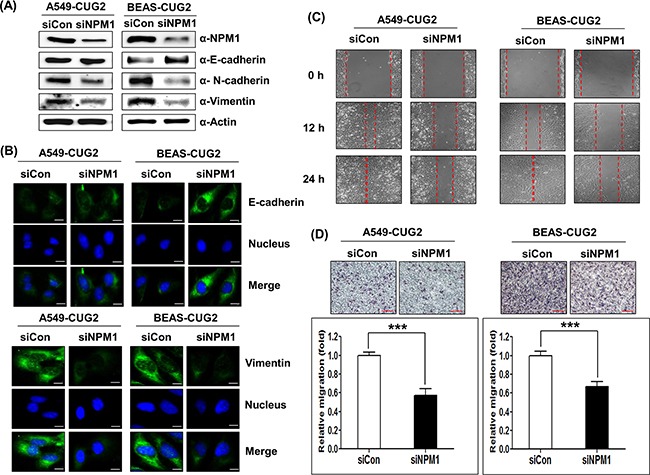
NPM1 silence inhibits the CUG2-induced EMT **A**. At 48 h post-treatment with NPM1 siRNA (500 nM), expression of NPM1, E-cadherin, N-cadherin, and vimentin in A549-CUG2 and BEAS-CUG2 cells was detected by immunoblotting. (siCon; control siRNA, siNPM1; NPM1 siRNA) **B**. A549-CUG2 and BEAS-CUG2 cells were incubated on chamber slide followed by fixation and permeabilization at 48 h post-treatment with NPM1 siRNA(500 nM), Expression of E-cadherin and vimentin was detected by immunofluorescence using Alexa Fluor 488-conjugated goat anti-mouse IgG (green) and Alexa Fluor 488-conjugated donkey anti-goat IgG (green), respectively. For nuclear staining, DAPI was added prior to mounting in glycerol. Scale bar indicates 10 μm. **C**. Cell migration was measured by a wound healing assay in A549- CUG2 and BEAS-CUG2 cells at 48 h post-treatment with NPM1 siRNA. The wound closure areas were monitored by phase-contrast microscopy at a magnification of 100×. The assays were repeated twice. **D**. An invasion assay was performed with A549-CUG2 and BEAS-CUG2 at 48 h post-treatment with NPM1 siRNA. Scale bar indicates 100 μm. The assays were repeated twice. Each assay was performed in triplicate and error bars indicate SD (***; *p*< 0.001).

Next, we wondered if suppression of endogenous NPM1 with its siRNA inhibits the CUG2-mediated EMT. We optimized NPM1 siRNA concentration to efficiently decrease NPM1 expression (Supplementary Figure S2). NPM1 suppression recovered E-cadherin expression and diminished levels of N-cadherin and vimentin protein in A549-CUG2 and BEAS-CUG2 cells compared to control siRNA treatment (Figure 2A), which was verified by immunofluorescence (Figure 2B). Eventually, NPM1 suppression decreased wound healing and tumor invasion capability of A549-CUG2 and BEAS-CUG2 cells (Figures 2C and 2D). Taken together, these results indicate that CUG2 induces the EMT, which requires interaction with NPM1.

### CUG2-induced EMT occurs via TGF-β signaling

**Figure 3:**
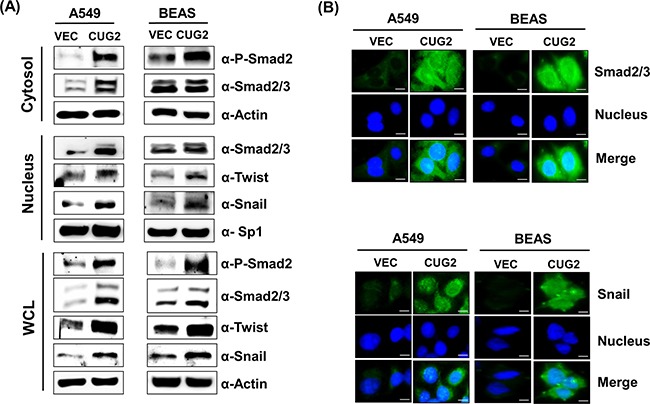
Overexpression of CUG2 activates TGF-β signaling **A**. Expression of phospho-Smad2, Smad2/3, Snail and Twist in A549-CUG2 and BEAS-CUG2 cells was compared with those in their control cells by immunoblotting. In addition, the cells were fractionated into cytosolic and nuclear extracts. Expression of the same proteins was detected by immunoblotting. Sp1 and actin were used loading controls for nuclear and cytosolic extracts, respectively. **B**. A549-Vec, A549-CUG2, BEAS-Vec and BEAS-CUG2 cells were incubated on chamber slide followed by fixation and permeabilization. Expression of Smad2/3 or Snail was detected by immunofluorescence using Alexa Fluor 488-conjugated goat anti-rabbit IgG (green). For nuclear staining, DAPI was added prior to mounting in glycerol. Scale bar indicates 10 μm.

**Figure 4:**
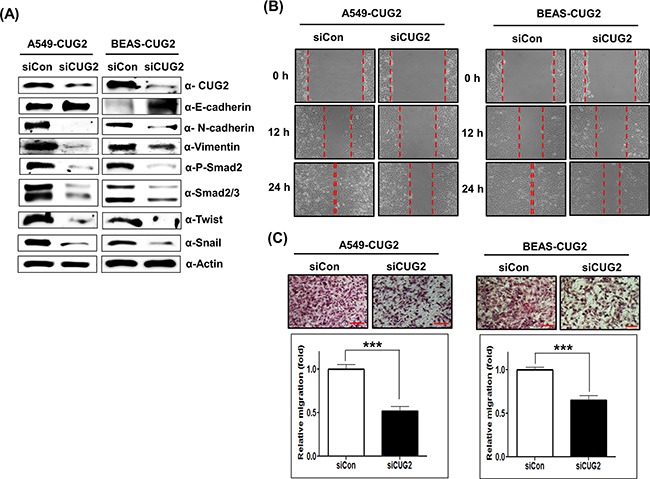
CUG2 silence inhibits TGF-β signaling, leading to suppression of the EMT **A**. At 48 h post-treatment with CUG siRNA (500 nM), expression of CUG2, E-cadherin, N-cadherin, vimentin, phospho-Smad2, Smad2/3, Snail and Twist in A549-CUG2 and BEAS-CUG2 cells was detected by immunoblotting. (siCon; control siRNA, siCUG2; CUG2 siRNA). **B**. Cell migration was measured by a wound healing assay in A549- CUG2 and BEAS-CUG2 cells at 48 h post-treatment with CUG2 siRNA. The wound closure areas were monitored by phase-contrast microscopy at a magnification of 100×. The assays were repeated twice. **C**. An invasion assay was performed with A549-CUG2 and BEAS-CUG2 at 48 h post-treatment with NPM1 siRNA. Scale bar indicates 100 μm. The assays were repeated twice. Each assay was performed in triplicate and error bars indicate SD (***;*p*< 0.001).

Since it was reported that TGF-β signaling plays a crucial role in inducing the expression of several acting transcription factors such as Snail, Slug and Twist, which are known as ‘master regulators’ of the EMT [12, 13], we wondered whether overexpression of CUG2 induces up-regulation of TGF-β signaling proteins. We then found that A549-CUG2 and BEAS-CUG2 cells experienced an increase of Smad2/3 protein levels and activation of Smad2 in the cytoplasm compared to A549-Vec and BEAS-Vec cells, leading to translocation of Smad2/3 to the nucleus (Figures 3A and 3B). We also observed higher expression of Snail and Twist in the nucleus of A549-CUG2 and BEAS-CUG2 cells compared to those in A549-Vec and BEAS-Vec cells (Figures 3A and 3B). Conversely, suppression of CUG2 expression with its siRNA slightly recovered E-cadherin expression in A549-CUG2 cells, but fully recovered E-cadherin protein levels in BEAS-CUG2 cells. Expression of N-cadherin and vimentin levels was decreased during the CUG2 silence (Figure 4A). The CUG2 silence also decreased Smad2/3, Snail, and Twist protein levels in A549-CUG2 and BEAS-CUG2 cells (Figure 4A). Consequently, CUG2 siRNA treatment significantly inhibited wound healing and cell invasion in A549-CUG2 and BEAS-CUG2 cells compared to control siRNA treatment (Figures 4B and 4C). These results indicate that CUG2 overexpression up-regulates TGF-β signaling, leading to induction of EMT.

To confirm the positive role of TGF-β in the EMT, A549-Vec and BEAS-Vec cells were treated with TGF-β1, and then the protein levels of E-cadherin, N-cadherin and vimentin were examined. TGF-β1 treatment decreased E-cadherin protein levels and increased expression of N-cadherin and vimentin protein in both A549-Vec and BEAS-Vec cells (Supplementary Figure S3A). These changes led to rapid wound healing and cell invasion (Supplementary Figures S3B and S3C). We confirmed that TGF-β1 treatment induces activation of Smad2/3, Snail, and Twist (Supplementary Figure S3D). The results support that TGF-β1 treatment alone is enough to induce EMT in A549-Vec and BEAS-Vec cells as seen in other studies [19, 20].

**Figure 5:**
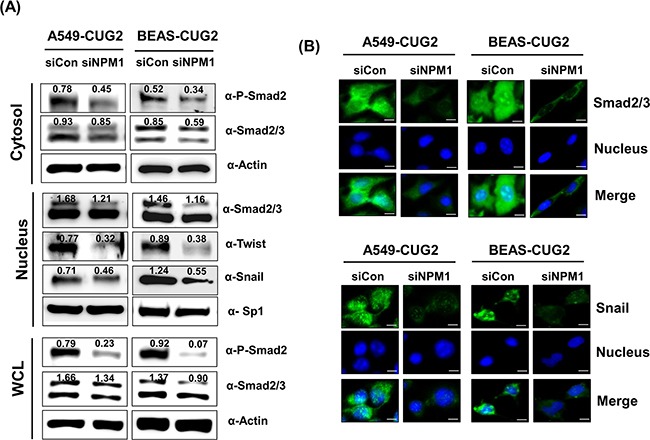
NPM1 silence inhibits TGF-β signaling **A**. At 48 h post-treatment with NPM1 siRNA (500 nM), A549-CUG2 and BEAS-CUG2 cells were fractionated into cytosolic and nuclear extracts. The whole cell lysates were also prepared at 48 h post-treatment with NPM1 siRNA. Expression of phospho-Smad2, Smad2/3, Snail and Twist was detected by immunoblotting. Sp1 and actin were used loading controls for nuclear and cytosolic extracts, respectively Image intensity was analyzed using ImageJ program (http://rsbweb.nih.gov./ij/plugins). **B**. A549-CUG2 and BEAS-CUG2 cells were incubated on chamber slide followed by fixation and permeabilization at 48 h post-treatment with NPM1 siRNA (500 nM). Expression of Smad2/3 or Snail was detected by immunofluorescence using Alexa Fluor 488-conjugated goat anti-rabbit IgG (green). For nuclear staining, DAPI was added prior to mounting in glycerol. Scale bar indicates 10 μm.

Moreover, to determine whether NPM1 is also involved in the activation of CUG2-mediated TGF-β signaling, we suppressed NPM1 protein levels using NPM1-specific siRNA. When NPM1 protein levels were decreased, Smad2/3 protein levels were also reduced in the whole cell lysates (Figure 5A). NPM1 suppression decreased cytoplasmic levels of phospho-Smad2 and Smad2/3 expression, which consequently inhibited translocation of Smad2/3 into the nucleus (Figures 5A and 5B). Snail and Twist expression also decreased in the nucleus during NPM1 suppression (Figures 5A and 5B). These results confirmed that NPM1 is involved in CUG2-mediated TGF-β signaling, leading to the induction of EMT. Taken together, we propose that TGF-β signaling is involved in CUG2-induced EMT.

### Inhibition of TGF-β signaling suppresses the CUG2-induced EMT

**Figure 6:**
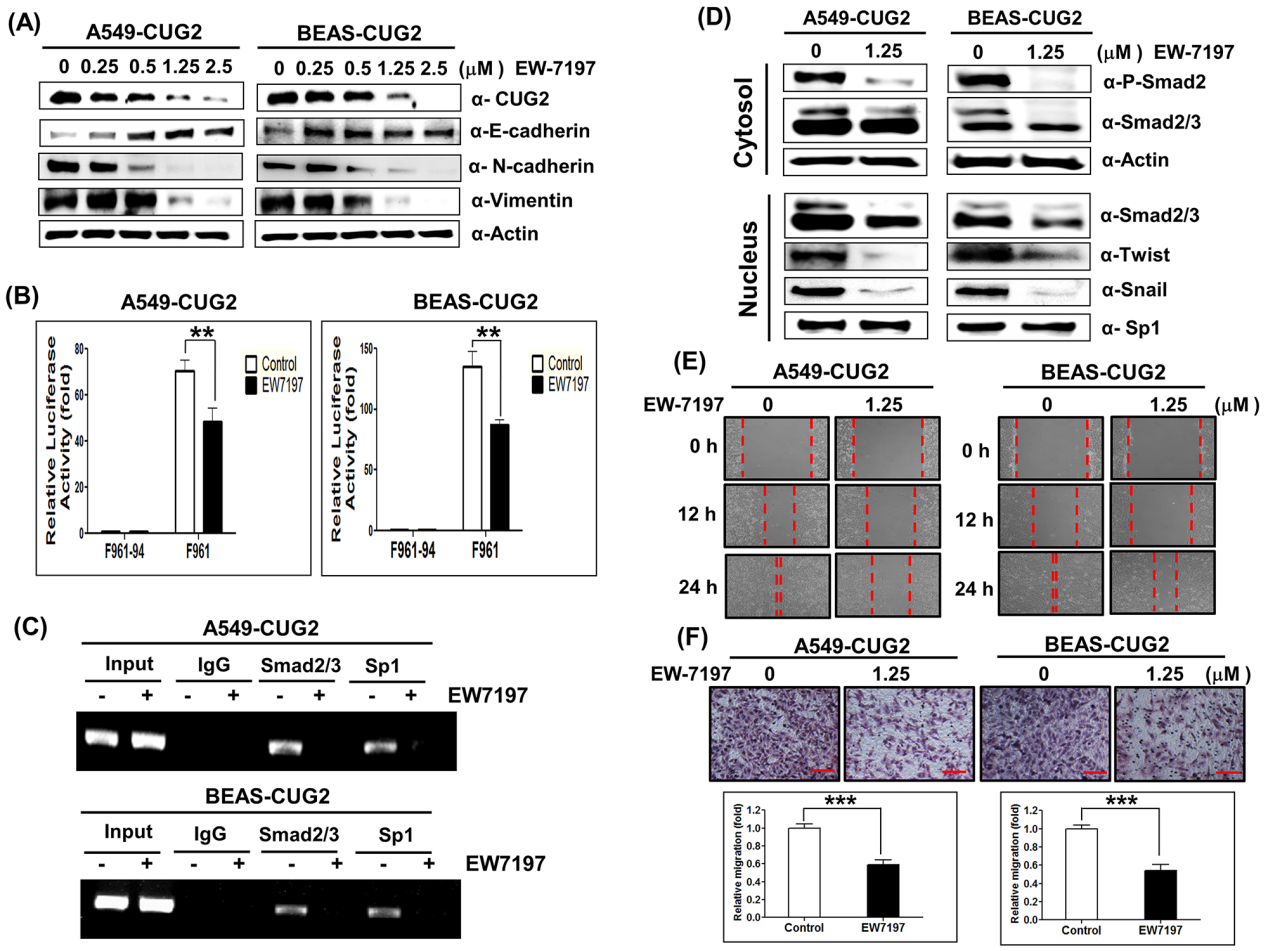
Treatment with EW-7197 inhibits the CUG2-induced EMT **A**. A549-CUG2 and BEAS-CUG2 cells were treated with EW-7197 at different doses (0.25, 0.5, 1.25 and 2.5 μM) for 24 h. Expression of CUG2, E-cadherin, N-cadherin, and vimentin was detected by immunoblotting. **B**. A549-CUG2 or BEAS-CUG2 cells were transfected with CUG2 promoter vectors (F961 and F961-94). At 48 h post-transfection, luciferase enzyme activities were measured in the transfected cell lysates. Transfection efficiency was normalized with the β-galactosidase reporter vector, pGK-β-gal. The assays were repeated in triplicate. The results shown are the average of triplicate wells. Error bars indicate SD. (**; *p*< 0.01) **C**. ChIP assays were performed with A549-CUG2 and BEAS-CUG2 cells. Chromatin fragments were pulled down with anti-Sp1, Smad2/3 antibodies or IgG as a control. Semi-quantitative PCRs were performed using specific CUG2 promoter primers. The assay was repeated twice. **D**. Expression of phospho-Smad2, Smad2/3, Snail and Twist in A549-CUG2 and BEAS-CUG2 cells treated with EW-7197 was detected by immunoblotting after cellular fractionations. Sp1 and actin were used loading controls for nuclear and cytosolic extracts, respectively. **E**. Cell migration was measured in A549-CUG2 and BEAS-CUG2 cells treated with EW-7197 by a wound healing assay. The assays were repeated twice. **F**. An invasion assay was performed with A549-CUG2 and BEAS-CUG2 cells treated with EW-7197. Scale bar indicates 100 μm. The assays were repeated twice. Each assay was performed in triplicate and error bars indicate SD. (***; *p*< 0.001).

To directly address whether TGF-β signaling plays a crucial role in the CUG2-mediated EMT, we treated A549-CUG2 and BEAS-CUG2 cells with EW-7197 [N-((4-([1,2,4]-triazolo[1,5-a]pyridin-6-yl)-5-(6-methylpyridin-2-yl)-1H-imidazol-2-yl)-methyl)-2-fluoroaniline], a novel activin receptor-like kinase 5 (ALK5) inhibitor [21]. Since it was reported that ALK5 inhibitors block phosphorylation of R-Smads by occupying the ATP binding site in the domain of TGF-β receptor I, EW-7197 was used for inhibition of TGF-β signaling [22]. When A549-CUG2 and BEAS-CUG2 cells were treated with EW-7197 at different concentrations, E-cadherin protein levels recovered in a dose-dependent manner in A549-CUG2 cells but simply recovered in BEAS-CUG2 cells (Figure 6A). N-cadherin and vimentin protein levels gradually decreased in a dose-dependent manner in A549-CUG2 and BEAS-CUG2 cells (Figure 6A). In contrast to TGF-β treatment, EW-7197 treatment gradually reduced CUG2 protein levels in a dose-dependent manner (Figure 6A). To investigate a further mechanism by which TGF-β inhibition suppresses CUG2 expression, we first explored the possibility that EW-7197 treatment enhances destabilization of CUG2 protein through proteasome-mediated degradation. To explore the hypothesis, MG132, a proteasome inhibitor, was introduced before the harvest of A549-CUG2 and BEAS-CUG2 cells treated with EW-7197. MG132 treatment did not block the decrease of CUG2 protein induced by EW-7197 treatment (Supplementary Figure S4A). This result indicates that EW-7197 does not induce CUG2 protein degradation through proteasome activity. Next, we examined CUG2 promoter activity with Sp1-binding sites using F961 luciferase reporter vector [23]. EW-7197 treatment significantly reduced the luciferase activity of the F961 vector compared to DMSO treatment as a control (Figure 6B). However, EW-7197 treatment did not affect the luciferase activity of CUG2 promoter without Sp1-binding sites using F961-34 luciferase reporter vector (Figure 6B). Additionally, to confirm that EW-7197 treatment inhibits synthesis of CUG2 transcripts as direct evidence, real-time quantitative RT-PCR (qRT-PCR) was performed. A549-CUG2 and BEAS-CUG2 treated with EW-7197 showed less abundant CUG2 transcripts than did the cells treated with DMSO as a control (Supplementary Figure S4B). These results suggest that inhibition of TGF-β signaling diminishes CUG2 expression at the transcriptional level. To illustrate the results, we used ChIP assay to examine whether EW-7197 treatment hinders Sp1 binding at the CUG2 promoter sites, leading to decreased CUG2 expression. After DMSO treatment, DNA fragments immunoprecipitated by Sp1 antibody were amplified by specific primers of CUG2 promoters. However, we could not do the same in the cells treated with EW-7197 (Figure 6C), indicating that TGF-β inhibition hinders Sp1 binding at CUG2 promoter sites. Since our previous study showed that binding of Sp1 to the CUG2 promoter is essential for CUG2 expression [23], this result of ChIP assay suggests that TGF-β signaling recruits Sp1 transcription factor to the CUG2 promoter, eventually leading to increased CUG2 protein levels. Of interest, we found that Smad2/3 protein interacts with the CUG2 promoter in A549-CUG2 and BEAS-CUG2 cells whereas EW-7197 treatment inhibits Smad2/3 binding to the CUG promoter (Figure 6C). EW-7197 also diminished phospho-Smad2 levels in the cytoplasm, which led to suppressed translocation of Smad2. EW-7197 inhibited expression of Snail protein in the nucleus compared to DMSO treatment (Figure 6D). Consequently, treatment with EW-7197 eventually inhibited wound healing and cell invasion in both A549-CUG2 and BEAS-CUG2 cells (Figures 6E and 6F). Moreover, when A549-CUG2 and BEAS-CUG2 cells were treated with TGF-β1 siRNAs, the cells exhibited higher expression of E-cadherin compared to control siRNA-treated cells (Supplementary Figure S5A). TGF-β1 siRNA treatment reduced N-cadherin and vimentin expression in A549-CUG2 and BEAS-CUG2 cells compared to control siRNA treatment (Supplementary Figure S5A). Consequently, TGF-β1 siRNA treatment inhibited CUG2-mediated cell wound healing and invasion compared to control siRNA treatment (Supplementary Figure S5B and S5C). These results confirmed that TGF-β signaling plays a critical role in the CUG2-mediated EMT.

### Akt and MAPKs are involved in the CUG2-induced EMT

**Figure 7:**
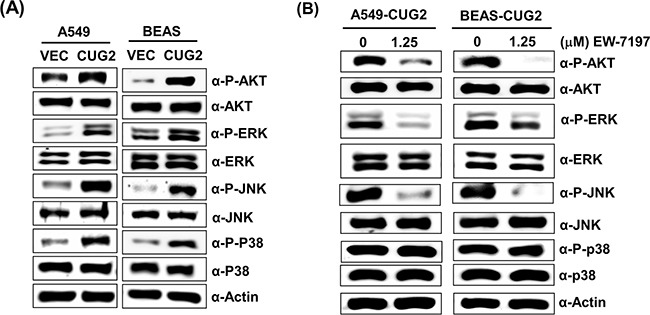
CUG2 activates Akt and MAPKs, which are dependent on TGF-β signaling, except p38 MAPK **A**. Activation of Akt, ERK, JNK and p38 MAPK in A549-CUG2 and BEAS-CUG2 cells was compared with those in their control cells by immunoblotting using their corresponding phospho-specific antibodies. **B**. A549-CUG2 and BEAS-CUG2 cells were treated with EW-7197 at the dose (1.25 μM) for 24 h. Activation of Akt, ERK, JNK and p38 MAPK was examined by immunoblotting.

**Figure 8:**
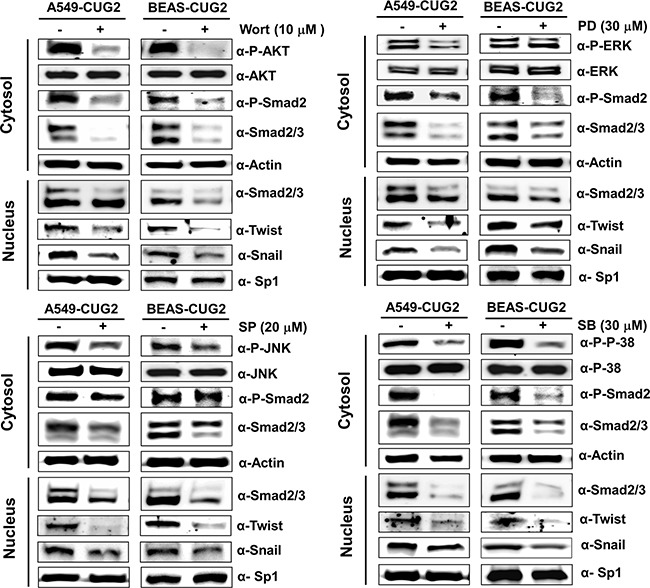
Treatment with Akt or MAPK inhibitors diminishes the CUG2-mediated TGF-β signaling After A549-CUG2 and BEAS-CUG2 cells were treated with wortmannin (Wort; 10 μM), PD98059 (PD; 30 μM), SP600125 (SP; 20 μM), or SB203580 (SB; 30 μM) for 24 h, inactivation of Akt, ERK, JNK and p38 MAPK was confirmed by immunoblotting using their corresponding phospho-specific antibodies. Expression of phospho-Smad2, Smad2/3, Snail and Twist was detected by immunoblotting after cellular fractionations. Sp1 and actin were used loading controls for nuclear and cytosolic extracts, respectively.

**Figure 9:**
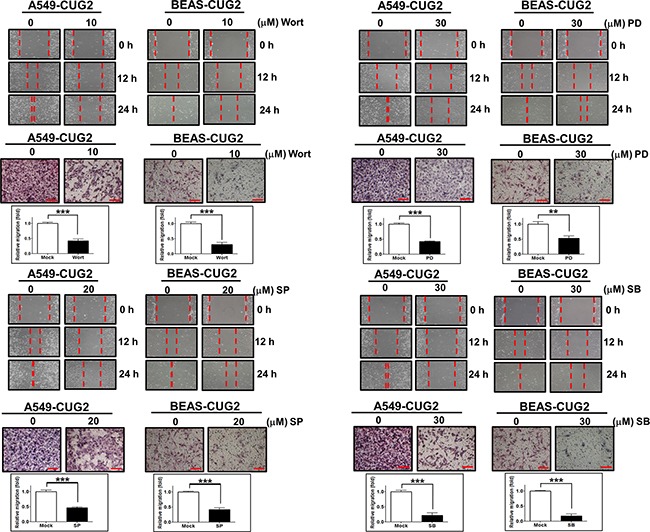
Inhibition of Akt and MAPKs hinders the CUG2-induced EMT A549-CUG2 and BEAS-CUG2 cells were treated with wortmannin (Wort), PD98059 (PD), SP600125 (SP), or SB203580 (SB). A wound healing assay was performed for cell migration. The wound closure areas were monitored by phase-contrast microscopy at a magnification of 100×. The assay was repeated twice. Cell invasion assay was performed using 48-well Boyden chambers. The chamber was assembled using polycarbonate filters coated with Matrigel. Scale bar indicates 100 μm. The assay was repeated twice. Each assay was performed in triplicate. Error bar indicates SD. All ***; *p* <0.001, except **; *p*<0.01 in BEAS-CUG2 cells treated with PD.

Since it was reported that TGF-β activates the Ras-ERK and TRAF6-TAK-p38 MAPK/JNK signaling pathways as non-canonical pathways [16, 17] and that CUG2 activated not only ERK but also p38 MAPK and JNK in a murine cell line from our previous study [4], we wondered whether inhibition of these signaling pathways decreases the CUG2-mediated EMT. We first confirmed activation of Akt, ERK, JNK and p38 MAPK in A549-CUG2 and BEAS-CUG2 cells (Figure 7A). We also wondered whether activation of Akt, ERK, JNK and p38 MAPK in A549-CUG2 and BEAS-CUG2 cells is dependent on TGF-β signaling pathway. To answer this question, A549-CUG2 and BEAS-CUG2 cells were treated with EW-7197, and we examined the phosphorylation levels of the kinases. We then found that EW-7197 treatment inhibits activation of Akt, ERK and JNK but fails to block activation of p38MAPK. These results indicate that activation of Akt, ERK and JNK is dependent on TGF-β signaling in A549-CUG2 and BEAS-CUG2 cells while activation of p38MAPK is independent of TGF-β signaling (Figure 7B). Based on these results, we expect that CUG2 activates Akt, ERK, and JNK through TGF-β signaling, but CUG2 harbors not only TGF-β but also another signaling pathway to activate p38MAPK. When A549-CUG2 and BEAS-CUG2 cells were treated with inhibitors of Akt, ERK, JNK, and p38 MAPK, levels of proteins downstream of TGF-β, including Smad2/3, Snail, and Twist were decreased (Figure 8), which led to suppression of wound healing and cell invasion in A549-CUG2 and BEAS-CUG2 cells (Figure 9). Additionally, when A549-Vec and BEAS-Vec cells were treated with these inhibitors, wound healing and cell invasion were reduced compared to those in DMSO-treated A549-Vec and BEAS-Vec cells. However, the inhibition levels of wound healing and cell invasion were not drastic compared to those in A549-CUG2 and BEAS-CUG2 treated with the inhibitors (Supplementary Figure S6), which may be attributed to a low endogenous expression of CUG2 in A549-Vec and BEAS-Vec cells. Taken together, the results suggest that activation of Akt and MAPKs plays important roles in the CUG2-mediated EMT, in which activation of Akt, ERK, and JNK is dependent on TGF-β signaling but activation of p38 MAPK is independent of TGF-β signaling

### Sp1 transcription factor collaborates with Smad2/3 proteins in the CUG2-induced EMT

**Figure 10:**
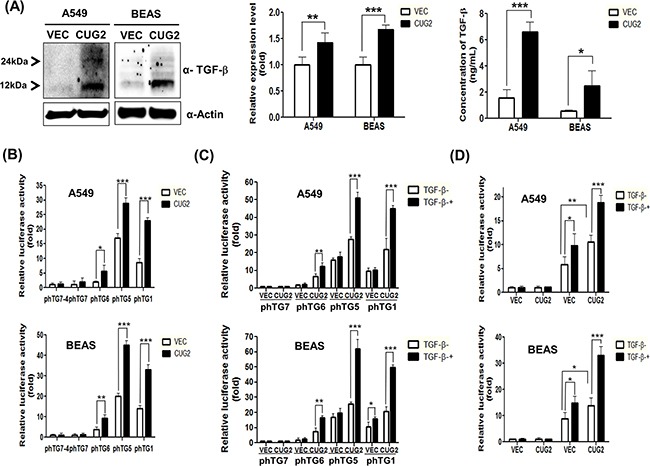
Sp1 transcription factor is required for both CUG2 and TGF-β transcription **A**. Production of TGF-β protein was detected with immunoblotting with an anti-TGF-β antibody after running of SDS-PAGE under a reduced condition. Production of TGF-β1 protein in the culture media from A549-CUG2 and BEAS-CUG2 cells was compared to that from their control cell media with a modified sandwich ELISA using Au nanoparticles. The assay was repeated in triplicate. The results shown are the average of triplicate wells and error bars indicate SD. (*; *p*< 0.05, ***; *p*< 0.001). Expression of TGF-β1 transcripts from A549-CUG2 and BEAS-CUG2 cells was compared to that from their control cells with qRT-PCR. The assay was repeated triplicate. Each assay was performed in triplicate and error bars indicate SD. (**; *p*< 0.01, ***; *p*< 0.001) **B-D**. A549-Vec, A549-CUG2, BEAS-Vec, or BEAS-CUG2 cells were transfected with TGF-β promoter vectors (phTG1, 5, 6, 7, and 7-4) or CUG2 promoter vectors (F961 and F961-94) in the absence and presence of TGF-β (5 ng/mL). At 48 h post-transfection, luciferase enzyme activities were measured in the transfected cell lysates. Transfection efficiency was normalized with the β-galactosidase reporter vector, pGK-β-gal. The assay was repeated in triplicate. The results shown are the average of triplicate wells. Error bars indicate SD. (*; *p*< 0.05,**; *p*<0.01, ***; *p*< 0.001)

Other studies have shown that Sp1 functions to mediate activation of the TGF-β promoter through canonical Sp1-binding sites for transcription of TGF-β [24], and also interacts with Smad2/3 for TGF-β-mediated up-regulation of α(1)(I)-collagen [25] and other genes [26]. Moreover, our previous study showed that the Sp1 transcription factor plays a critical role in CUG2 expression [23]. Based on these lines of evidence, we hypothesized that CUG2 activates TGF-β production via Sp1 that is recruiting Smad2/3 and conversely, TGF-β delivers signals to recruit Smad2/3 and Sp1 to the CUG2 promoter, which is essential for CUG2 expression. To test this possibility, we examined whether CUG2 overexpression results in TGF-β production, and then found that it indeed elevated TGF-β expression in both A549 and BEAS-2B cells (Figure 10A). To confirm that CUG2 overexpression increases TGF-β protein levels through the enhanced synthesis of TGF-β transcripts, we performed qRT-PCR. We found that A549-CUG2 and BEAS-CUG2 cells show more abundant TGF-β1 transcripts than their control cells (Figure 10A). Next, we attempted to detect secreted TGF-β1 from A549-CUG2 cells and BEAS-CUG2 cells in the medium, where it can be used in a paracrine or an autocrine manner. Herein, we introduced a new and verified ELISA method using gold nanoparticles which can enhance sensitivity to detect TGF-β1 as described elsewhere [27]. We found that A549-Vec cells intrinsically produce more TGF-β1 than BEAS-Vec cells. More importantly, A549-CUG2 cells and BEAS-CUG2 cells exhibited significantly more TGF-β1 production than their control cells (Figure 10A). To examine whether Sp1 transcription factor is involved in CUG2-mediated production of TGF-β, TGF-β promoter vectors with or without Sp1-binding sites were introduced. We then found that CUG2 expression increased luciferase activity of TGF-β promoter containing Sp1-binding sites [28] (phTG1, phTG5, and phTG6 reporter vectors) (Figure 10B). However, CUG2 expression failed to increase the luciferase activity of the TGF-β promoter lacking Sp1-binding sites (phTG7 and phTG7-4 reporter vectors) (Figure 10B). In addition, TGF-β1 treatment synergistically increased the CUG2-mediated luciferase activity in the phTG1 and phTG5 reporter vectors but not in the phTG7 reporter vectors (Figure 10C). The phTG6 lacking an AP1-binding site [28] showed a slight increase of luciferase activity after treatment with TGF-β1 (Figure 10C). In addition, to examine whether TGF-β1 enhances luciferase activity through Sp1-binding sites in the CUG2 promoter, A549-Vec and A549-CUG2 cells were treated with TGF-β1 after transfection with the F961 reporter vector or the F961-94 reporter vector [23]. Overexpression of CUG2 itself enhanced luciferase activity even in the absence of TGF-β1 after transfection with F961 reporter vector but failed to increase luciferase activity after transfection with F961-94 reporter vector (Figure 10D). TGF-β1 treatment in A549-Vec and BEAS-Vec cells elevated luciferase activity in the F961 reporter vector but not in the F961-94 reporter vector (Figure 10D), suggesting that Sp1 binding is required for TGF-β-mediated CUG2 expression. Similarly, a synergistic effect of TGF-β on CUG2 promoter activity was observed in A549-CUG2 and BEAS-CUG2 cells compared to that in A549-Vec and BEAS-Vec cells (Figure 10D). Furthermore, TGF-β1 treatment induced an increase in endogenous CUG2 protein levels (Supplementary Figure S3A), whereas inhibition of TGF-β signaling reduced the expression of endogenous CUG2 protein (Figure 6A). Based on these lines of evidence, we suggest that both CUG2-mediated TGF-β production and TGF-β-mediated CUG2 expression require Sp1 binding to their promoters.

**Figure 11:**
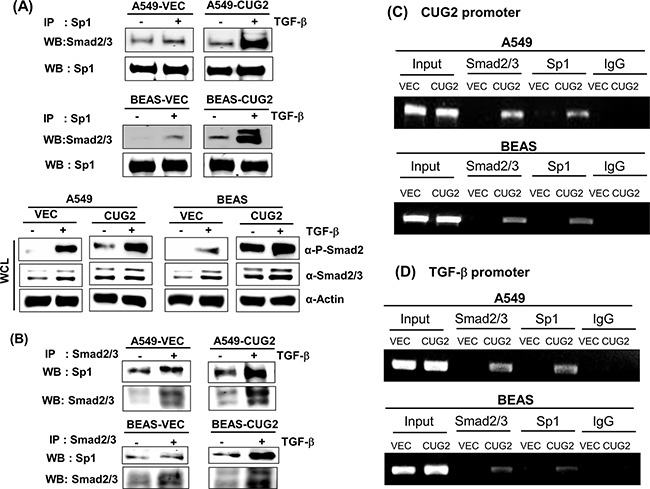
Interaction between Sp1 and Smad2/3 is involved in the synthesis of both CUG2 and TGF-β transcripts **A**. Sp1 proteins were pulled down from lysates of A549-Vec, A549-CUG2, BEAS-Vec, and BEAS-CUG2 cells in the absence or presence of TGF-β1 using an anti-Sp1 antibody. Smad2/3 proteins from the immunoprecipitates were detected with an anti-Smad2/3 antibody. **B**. Smad2/3 proteins were pulled down from lysates of A549-Vec, A549-CUG2, BEAS-Vec, and BEAS-CUG2 cells in the absence or presence of TGF-β1 using an anti-Smad2/3 antibody. Sp1 proteins from the immunoprecipitates were detected with an anti-Sp1 antibody. **C, D**. ChIP assays were performed with A549-Vec, A549-CUG2, BEAS-Vec, and BEAS-CUG2 cells. Chromatin fragments were pulled down with anti-Sp1, Smad2/3 antibodies or IgG as a control. Semi-quantitative PCRs were performed using specific CUG2 or TGF-β promoter primers. The assay was repeated twice.

Next, we investigated whether CUG2 expression induces Sp1 to interact with Smad2/3 for the synergistic effect of TGF-β. TGF-β1 treatment enhanced Smad2/3 protein levels in the whole cell lysates from A549-Vec and A549-CUG2 cells (Figure 11A). When Sp1 proteins were pulled down and then Smad2/3 proteins were examined in the immunoprecipitates by an anti-Smad2/3 antibody using these cell lysates, Smad2/3 proteins were found more abundantly in the immunoprecipitates from TGF-β1-treated A549-Vec and A549-CUG2 cells compared to those from the untreated cells (Figure 11A). Elevated expression of CUG2 alone also facilitated a slight increase in Smad2/3 binding to Sp1 in the immunoprecipitates (Figure 11A). Similar results were obtained in BEAS-Vec and BEAS-CUG2 cells (Figure 11A). Conversely, when Smad2/3 proteins were pulled down, Sp1 proteins were more abundantly detected in the immunoprecipitates of the TGF-β1-treated A549-CUG2 and BEAS-CUG2 cells compared to those from the untreated cells (Figure 11B). Moreover, to directly address whether Smad2/3 proteins are actively involved in the expression of CUG2 and whether Sp1 protein is closely associated with production of TGF-β, ChIP assays were performed. As seen in Figure 11C, Smad2/3 proteins were found together with Sp1 protein in the CUG2 promoters of A549-CUG2 and BEAS-CUG2 cells but not found in the promoters of A549-Vec and BEAS-Vec cells. When binding of Sp1 to the TGF-β promoter was examined, Sp1 protein was found together with Smad2/3 in A549-CUG2 and BEAS-CUG2 cells, but not in A549-Vec and BEAS-Vec cells (Figure 11D). Taken together, these results indicate that both Sp1 and Smad2/3 play crucial roles as mediators of the crosstalk between CUG2 and TGF-β.

### CUG2 and NPM1 are partially involved in TGF-β-mediated EMT

**Figure 12:**
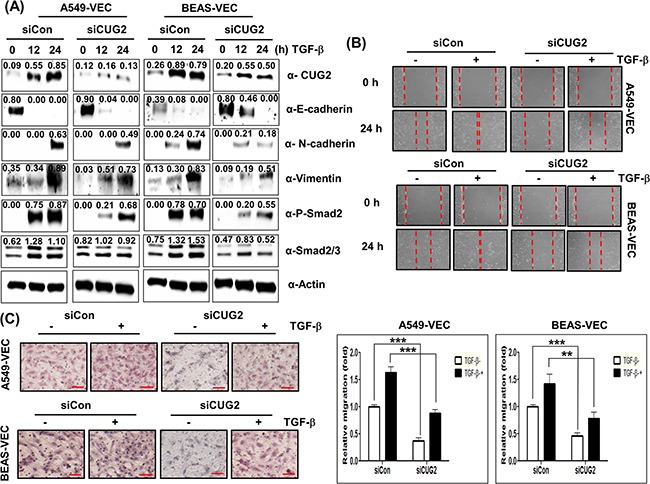
CUG2 is partially involved in TGF-β-mediated EMT **A**. A549-Vec and BEAS-Vec cells were treated with CUG2 siRNA (500 nM) prior to administration of TGF-β1 (5 ng/mL) for 24 h. Intracellular expression of CUG2, E-cadherin, N-cadherin, vimentin, phospho-Smad2 and Smad2/3 was detected by immunoblotting. Image intensity was analyzed using ImageJ program (http://rsbweb.nih.gov./ij/plugins). **B**. Cell migration was performed by a wound healing assay using A549-Vec and BEAS-Vec cells co-treated with CUG2 siRNA and TGF-β1. **C**. An invasion assay was performed with A549-Vec and BEAS-Vec cells co-treated with CUG2 siRNA and TGF-β1. Scale bar indicates 100 μm. The assay was repeated twice. Each assay was performed in triplicate, and error bars indicate SD. (**; *p*< 0.01, ***; *p*< 0.001).

Conversely, in order to determine whether CUG2 contributes to TGF-β-mediated EMT, we suppressed CUG2 expression with its siRNAs in the presence of TGF-β1. Suppression of CUG2 inhibited TGF-β1-mediated up-regulation of N-cadherin and vimentin expression in both A549-Vec and BEAS-Vec cells compared to control siRNA treatment (Figure 12A). The CUG2 siRNA treatment failed to block TGF-β1-mediated down-regulation of E-cadherin expression in A549-Vec cells, but successfully inhibited TGF-β1-mediated down-regulation of E-cadherin expression in BEAS-Vec cells (Figure 12A). Suppression of CUG2 inhibited TGF-β1-mediated up-regulation of Smad2/3 protein expression and phosphorylation of Smad2 in both A549-Vec and BEAS-Vec cells (Figure 12A). Consequently, the treatment with CUG2 siRNA inhibited TGF-β1-induced wound healing and invasion but did not completely block the TGF-β-induced EMT (Figures 12B and 12C). These results suggest that the effect of TGF-β on EMT is dominant over the effect of CUG2 on EMT and thus that CUG2 could still play a partial role in TGF-β-induced EMT.

**Figure 13:**
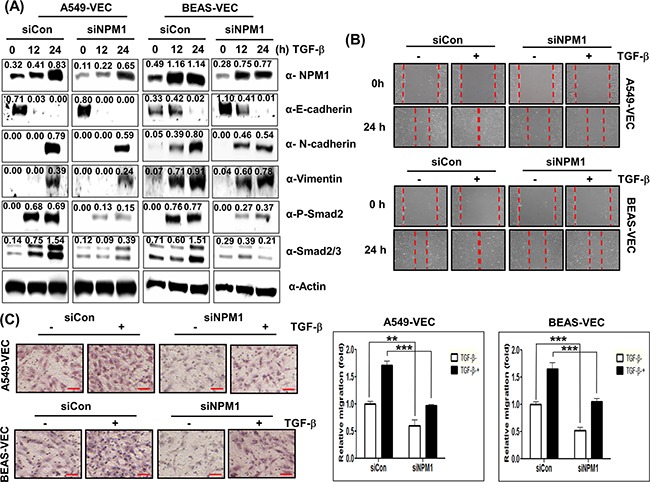
NPM1 is partially involved in TGF-β-mediated EMT **A**. A549-Vec and BEAS-Vec cells were treated with NPM1 siRNA (500 nM) prior to administration with TGF-β1 (5 ng/mL) for 24 h. Intracellular expression of NPM1, E-cadherin, N-cadherin, vimentin, phospho-Smad2 and Smad2/3 was detected by immunoblotting. Image intensity was analyzed using ImageJ program (http://rsbweb.nih.gov./ij/plugins). **B**. Cell migration was performed by a wound healing assay using A549-Vec and BEAS-Vec cells co-treated with NPM1 siRNA and TGF-β1. The assay was repeated twice. **C**. An invasion assay was performed with A549-Vec and BEAS-Vec cells co-treated with NPM1 siRNA and TGF-β1. Scale bar indicates 100 μm. The assay was repeated twice. Each assay was performed in triplicate, and error bars indicate SD. (**; *p*< 0.01, ***; *p*< 0.001).

Next, in order to examine whether NPM1 contributes to TGF-β-mediated EMT, we suppressed NPM1 expression with its siRNA in the presence of TGF-β1. Suppression of NPM1 inhibited TGF-β1-mediated up-regulation of N-cadherin and vimentin expression in both A549-Vec and BEAS-Vec cells while the treatment with NPM1 siRNA did not block TGF-β1-mediated down-regulation of E-cadherin expression (Figure 13A). Suppression of NPM1 diminished TGF-β1-mediated up-regulation of Smad2/3 protein expression and phosphorylation of Smad2 in A549-Vec and BEAS-Vec cells (Figure 13A). Consequently, the treatment with NPM1 siRNA inhibited TGF-β1-induced wound healing and invasion but did not completely block TGF-β1-induced EMT (Figures 13B and 13C). Since we observed that the effect of NPM1 on TGF-β1-induced EMT was almost the same result seen in the effect of CUG2 on TGF-β1-induced EMT, we wondered whether NPM1 plays a role in EMT independently of CUG2. Suppression of CUG2 resulted in a decrease in NPM1 protein levels and moreover, silence of NPM1 caused a decrease in CUG2 expression (Supplementary Figure S7), indicating that both CUG2 and NPM1 are complementary and interdependent for the effect on EMT. Taken together, these results indicate that the effect of TGF-β on EMT is dominant over the effect of NPM1 on EMT and thus that NPM1 could still play a partial role in TGF-β-induced EMT.

## DISCUSSION

Centromere proteins (CENPs) play a fundamental role in the cell cycle because assembly of CENP-A, a histone H-3 related protein into a constitutive centromere-associated network (CCAN) composed of 16 proteins CENP-C, -H, -I, -K, -L, -M, -L, -O, -P, -Q, -R, -U, -T, -W, -S, and -X, recruits outer kinetochore components, leading to attachment of spindle tubes [29]. From a clinical perspective, it has been suggested that deregulation of CENPs is involved in carcinogenesis. For instance, overexpression of CENP-A has frequently been found in lung adenocarcinoma cancer patients [30] and colorectal cancer tissues [31]. CENP -F was shown to be highly up-regulated in breast cancer samples and was identified as a new biomarker associated with poor outcome in these cancer patients [32, 33]. Enhanced expression of CENP-H was also detected in nasopharyngeal carcinoma [34] and colorectal cancers [35]. Further studies have shown that elevated expression of CENP-A induces genomic instability in pRb-depleted colon cancer cell [36], and that ectopic expression of CENP-H induces chromosomal missegregation and aneuploidy [35], suggesting a crucial role of CENPs as mediator during mitosis. We also showed that suppression of CUG2, known as CENP-W, induces chromosomal missegregation during cell division because of a failure of chromosomal linkage to the kinetochore complex [3]. However, we herein report another novel function of CENP-W besides its involvement in the cell cycle: up-regulated expression of CENP-W induces the EMT, a prelude to metastasis, through TGF-β signaling.

Moreover, we focused on the role of NPM1 in the CUG2-mediated EMT because we observed that loss of interaction between CUG2 and NPM1 leads to failure to induce the EMT (Figures 1 and 2). Because it was reported that NPM1 stabilizes CUG2 protein levels [18], we believe that this interaction continuously maintains a certain level of CUG2 protein in order to induce TGF-β production. Of course, because overexpression or mutation of NPM1 is frequently detected in human cancer tissues [37, 38], we wonder whether NPM1 expression is increased in A549-CUG2 and BEAS-CUG2 cells. We could detect the elevated expression of NPM1 in these cell lines as seen in Supplementary Figure S7. However, considering that NPM1 is also involved in ARF-P53 interaction [39], we cannot exclude the possibility that NPM1 contributes to the CUG2-mediated EMT through other signaling molecules.

**Figure 14:**
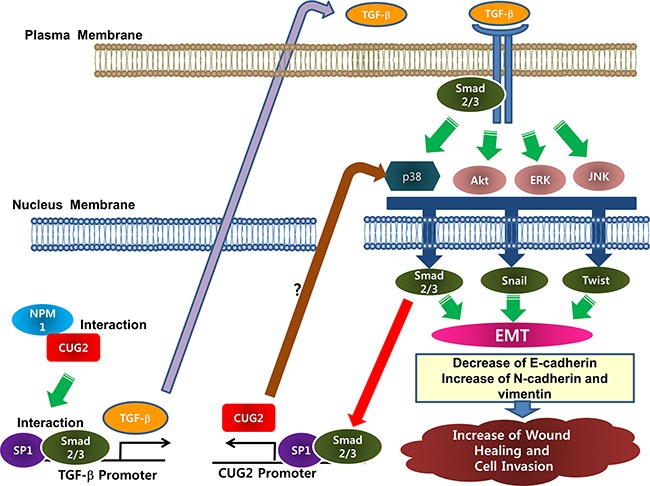
Schematic diagram of a mechanism by which CUG2 induces EMT Overexpression of CUG2 together with NPM1 activates Sp1 and Smad2/3 transcription factors, which bind to the TGF-β promoter. This eventually enhances production of TGF-β protein. The produced TGF-β binds to the TGF-β receptor in autocrine or paracrine manner. TGF-β signaling activates Akt and MAPKs in non-canonical ways. P38 MAPK is also directly activated by CUG2 although the signaling pathway is unknown. Subsequently, Akt and MAPK activate Smad2/3, Snail, Twist transcription factors as master regulators of EMT. Finally, Sp1 and activated Smad2/3 in the nucleus enhances binding to the CUG2 promoter, leading to increase of CUG2 transcription, which is a synergistic feedback system for both the elevated expression of CUG2 and TGF-β.

Eventually, we were curious how CUG2 induces TGF-β production, leading to the EMT. Our study showed that CUG2 induces activation of Akt and MAPKs including ERK, JNK, and p38 MAPK. Inhibition of these kinases hindered TGF-β signaling. Of interest, inhibition of TGF-β signaling with EW-7197 conversely suppressed activation of Akt, ERK and JNK but not the activation of p38 MAPK. Based on these results, we could suggest that CUG2 activates Akt, ERK and JNK through TGF-β signaling but CUG2 activates p38 MAPK in a TGF-β independent manner. Thus, an assignment to investigate the distinct regulation of MAPKs remains for the next study. We moreover found that TGF-β treatment enhances CUG2 expression (Supplementary Figure S3A). According to another line of evidence showing that Sp1 plays a crucial role in CUG2 expression [23], we imagine that the Sp1 transcription factor offers a clue to the connection between CUG2 and TGF-β. Our effort to link them led to evidence demonstrating that Sp1 binding is required for TGF-β transcription [24]. These results finally prompted us to attempt co-immunoprecipitation and ChIP assays to address Sp1 and Smad2/3 interactions, and Sp1 and Smad2/3 binding to the CUG2 and TGF-β promoters, which leads to cross-talk between CUG2 and TGF-β through Sp1 and Smad2/3 transcription factors (Figures 10 and 11). The results suggest that CUG2 and TGF-β synergistically induce the EMT by cooperation of Sp1 and Smad2/3, leading to metastasis of lung cancer cells. Based on all results shown in this study, we depicted a schematic diagram illustrating how overexpression of CUG2 induces EMT (Figure 14).

## MATERIALS AND METHODS

### Cell cultures

Human lung cancer A549 cells (ATCC, Manassas, VA) and immortalized human bronchial BEAS-2B cells (ATCC) stably expressing either vector alone (A549-Vec; BEAS-Vec), wild type CUG2 (A549-CUG2; BEAS-CUG2), the N-terminal domain (amino acids 1-66) of CUG2 (A549-CUG2NT; BEAS-CUG2NT), and the C-terminal domain (amino acid 31-88) of CUG2 (A549-CUG2CT; BEAS-CUG2CT) were cultured in RPMI-1640 and 50% DMEM/ 50% F12, respectively. These media were supplemented with 10% FBS, 1% penicillin, 1% streptomycin, and G418 (Sigma-Aldrich, St. Louis, MO; 1.5 mg/ml) at 37°C and 5% CO_2_.

### Reagents and antibodies

For immunoblotting, anti-AKT, -ERK, -JNK, -p38 MAPK, -Smad2/3 antibodies and their corresponding phospho-specific antibodies were acquired from Cell Signaling Biotechnology (Danvers, MA). Anti-β-actin, -NPM1, -Sp1 and -TGF-β antibodies were obtained from Santa Cruz Biotechnology (Santa Cruz, CA) and anti-E-cadherin, -N-cadherin, -vimentin, -Snail, -Twist and -CUG2 antibodies were obtained from Abcam (Cambridge, MA). For inhibition of protein kinases, wortmannin, PD98059, SP600125, and SB203580 were purchased from Calbiochem (San Diego, CA). TGF-β1 was obtained from R&D Systems (Minneapolis, MN). The TGF-β inhibitor EW-7197 was synthesized as described previously [21].

### Cellular fractionation

As described elsewhere [40], cells cultured in 100-mm plates were washed and harvested with ice-cold PBS and cell pellets were lysed with 800 μL of TTN buffer (20 mM Tris-HCl [pH7.4], 0.05% Triton-X100, 150 mM NaCl, 1 mM EDTA, 1 mM DTT, 10% glycerol, 0.5 mM PMSF, and 1x protease inhibitor cocktail) on ice for 20 min followed by centrifugation at 10,000 *g* for 15 min. The supernatant was taken as the soluble fraction, and the pellets as insoluble fractions were subsequently solubilized in 800 μL of RIPA buffer (50 mM Tris-HCl [pH7.4], 150 mM NaCl, 1 mM EDTA, 1 mM DTT, 1% NP-40, 0.5% deoxycholic acid, 0.1% SDS, 10% glycerol, 0.5 mM PMSF, and 1x protease inhibitor cocktail) on ice for 30 min and were centrifuged at 12,000*g* for 15 min. Thereafter, the supernatants were used for the nuclear extracts.

### Immunoblotting and Immunoprecipitation

Cells were harvested and lysed with lysis buffer containing 1% NP-40 and protease inhibitors (Sigma-Aldrich). For immunoblotting, proteins from whole cell lysates were resolved by 10% or 15% SDS-polyacrylamide gel electrophoresis (PAGE) and then transferred to nitrocellulose membranes. Primary antibodies were used at a 1:1000 or 1:2000 dilution, and secondary antibodies conjugated with horseradish peroxidase were used at a 1:2000 dilution in 5% nonfat dry milk. For immunoprecipitation, cells were harvested after 48 h of transfection, and the cell debris was removed by centrifugation at 10,000 *g* for 10 min at 4°C. Cell lysates were pre-cleared with 25 μL of protein A/G agarose and incubated with the appropriate primary antibody and protein A/G agarose for 1 h at 4°C. After 3 washes with lysis buffer, the precipitates were resolved on SDS-PAGE gels and analyzed by immunoblotting with the appropriate antibodies. After the final washing, the membranes were evaluated with an enhanced chemiluminescence assay using the Image Quant LAS 4000 Mini (GE-Healthcare, Tokyo, Japan).

### Luciferase reporter assays

A549-Vec, A549-CUG2, BEAS-Vec, and BEAS-CUG2 cells were transfected with TGF-β promoter vectors (phTG1, 5, 6, 7, and 7-4) [41], or CUG2 promoter vectors (F961 and F961-94)[23] with Lipofectamine 2000 (Invitrogen, Carlsbad, CA). To normalize transfection efficiency, a pGK-βgal vector that expresses β-galactosidase under a control of a phosphoglucokinase promoter was included in the transfection mixture. At 48 h post-transfection, cells were washed with cold PBS and lysed in lysis solution (25 mM Tris [pH7.8], 2 mM EDTA, 2 mM DTT, 10% glycerol, and 1% Triton-X100). Luciferase activity was measured with a luminometer by using a luciferase kit (Promega, Madison, WI).

### Short interfering RNA (siRNA) transfection

Cells were trypsinized and cultured overnight to achieve 60-70% confluency before siRNA transfection. NPM1 siRNAs (#1 ; AAC ACC ACC AGU GGU CUU AAG, # 2 ; GAA AAU GAG CAC CAG UUA U, Bioneer, Daejeon, Korea), pre-made CUG2 siRNA (Bioneer), TGF-β1 siRNA (Bioneer), or a negative control siRNA (Bioneer) were mixed with Lipofectamine 2000. The cells were incubated with the transfection mixture for 6 h and then rinsed with medium containing 10% FBS. The cells were incubated for 48 h before harvesting.

### Invasion assay

Invasion assays were performed using 48-well Boyden chambers (Neuroprobe, Gaithersburg, MD) as described elsewhere [42]. The lower wells of the chamber were filled with standard culture media. The chamber was assembled using polycarbonate filters (Neuroprobe) coated with Matrigel. Cells in serum-free media (5×10^4^ cells per well) were seeded in the upper compartment of the chamber. After incubation for 24 h, cell migration was quantified by counting the number of migrated cells after staining with hematoxylin-eosin.

### Wound healing assay

Cell migration was assessed using a scratch wound assay [43]. Briefly, the cells were cultured in six-well plates (5 × 10^5^ cells per well). When the cells were reached 90% confluence, a single wound was made in the center of the cell monolayer using a P-200 pipette tip. At 0 and 24 h of incubation, the wound closure areas were visualized by phase-contrast microscopy (Olympus, CKX31-11 PHP, Tokyo, Japan) at a magnification of 100x.

### Immunofluorescence

Cells were fixed with 4% paraformaldehyde for 15 min, permeabilized with cold acetone for 15 min, blocked with 10% goat serum for 30 min, and treated with primary antibodies (1:100 dilution) for 30 min at room temperature. After incubation, the cells were washed extensively with PBS, incubated with Alexa Fluor 418-conjugated goat anti-mouse or donkey anti-rabbit antibody (1:500 dilution; Molecular Probes, Eugene, OR) in PBS for 30 min at room temperature, and washed 3 times with PBS. For nuclear staining, the cells were incubated with DAPI for 5 min in the dark and washed 3 times with PBS. The stained cells were mounted using PBS containing 10% glycerol and were photographed using a fluorescence microscope (Zeiss, Axio Observer D1).

### Chromatin Immunoprecipitation (ChIP) assay

The ChIP assay was performed as described elsewhere [44]. A549-Vec, A549-CUG2, BEAS-Vec and BEAS-CUG2 cells growing on 10-cm tissue culture plates were cross-linked using 1% formaldehyde for 15 min at room temperature and quenched with glycine (125 mM). The cells then were harvested with SDS lysis buffer, and sheared by sonication to generate 300- to 800-bp fragments of DNA. Immunoprecipitation was performed with 4 μg of specific antibody at 4°C overnight in a rotary shaker. The samples were further incubated with 25 μL of Protein A-Sepharose resin (Santa Cruz Biotechnology) for 2 h to capture the protein-DNA-antibody complexes and DNA fragments were eluted in TE buffer (10 mM Tris-HCl [pH8.0], 1 mM EDTA) by heating at 90°C for 10 min and analyzed by semi-quantitative PCR using CUG2 or TGF-β promoter specific primers. CUG2 promoter primers were as follows: sense 5′-AAC TTC CAA TCA TCT CTA GGG AAC C-3′, antisense 5′-CGT ATG ACG CTT CTT CAG GCA GAA-3′. TGF-β1 promoter primers were as follows: sense 5′-TAC CAG ATC GCG CCC ATC TAG-3′, antisense 5′-ACT GCC GAG AGC GCG AAC AG-3′.

### Real-time quantitative Reverse Transcription-Polymerase Chain Reaction (qRT-PCR)

Total RNA was isolated from cells and the cDNAs were synthesized using the QuantiTect Probe reverse transcriptase-PCR (RT-PCR) kit (Qiagen) according to the protocols provided by the manufacturer. Real time RT-PCR was conducted on a AriaMx Real-Time System (Agilent Technologies, Santa Clara, CA) using the SYBR Premix EX Taq (Takara, Kusatsu, Japan) and the following gene-specific primers: *TGF-β1* (forward) 5′ – CTT TGG TAT CGT GGA AGG ACT C- 3′; and (reverse) 5′ – AGC TGT ACC AGA AAT ACA GCA ACA - 3′; *CUG2* (forward) 5′-GAA GCC TCA ACT TCG TCT GG-3′; and (reverse) 5′-GTA GAG GCA GGG ATG ATG TTC T-3′. Real-time RT-PCR data were obtained in the form of threshold cycle (C*t*) values, and target gene expression was normalized to GADPH expression. Relative expression levels of target genes (*TGF-β1* and *CUG2*) were calculated by the comparative C*t* (2^−ΔΔCτ^) method as previously described [45, 46].

### Enzyme–linked immunosorbent assay (ELISA)

The ELISA described elsewhere [27] was slightly modified. The polystyrene 96-well plate was coated with TGF-β1 capture antibody (recognizing TGF-β1 C-terminus; Santa Cruz Biotech.). Cell medium supernatants were added to the capture antibody immobilized plate. The detection TGF-β1 antibody (recognizing whole TGF-β1; R&D Systems)-conjugated (+)Au nanoparticle (NP) solution was added to the plate and also bound with antigens through antigen-antibody reaction. Unbound (+)AuNPs were washed out. 3,3′,5,5′-tetramethylbenzidine (TMB)-H_2_O_2_ substrates were added and enzymatic reaction was occurred due to peroxidase-like activity of (+)AuNPs. After the reaction was ended by a stopping agent, O.D. value at 450 nm was measured.

### Statistical analysis

Data were presented as means ± standard deviation (SD). One-way Anova or Unpaired t test in GraphPad Prism was used for statistical analysis, with *p*-value of <0.05 defined as significant.

## SUPPLEMENTARY MATERIALS FIGURES AND TABLES


