# Supplementary material for: Cancer upregulated gene 2 induces epithelial-mesenchymal transition of human lung cancer cells via TGF-β signaling
Source: Oncotarget. 2016 Dec 10;8(3):5092–110. doi: 10.18632/oncotarget.13867 (PMC5354895; doi:10.18632/oncotarget.13867)
Supplement: Supplementary file 1 [file oncotarget-08-5092-s001.pdf]

Cancer upregulated gene 2 induces epithelial-mesenchymal transition of human lung cancer cells via TGF-β signaling

SUPPLEMENTARY FIGURES

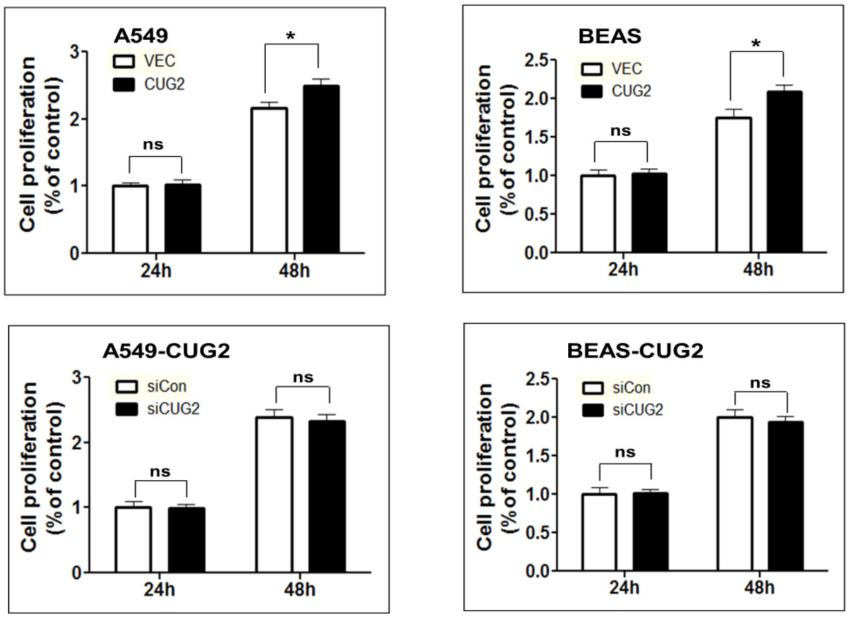

Supplementary Figure S1: No difference of cell growth between CUG2 overexpressing cells and their control cells during 24 h. A549-CUG2, BEAS-CUG2 and their control cells were inoculated at $1x10^5$ cells per well using a 12-well plate. We set 12 h post-inoculation as an initiation time for the measurement of cell growth. At 12 h post-inoculation, CUG2 siRNA (500 nM) or control siRNA (500 nM) was added. (siCon; Control siRNA, siCUG2; CUG2 siRNA) MTT assays were performed for measuring cell growth at 24 and 48 h. Dye solution containing tetrazolium was added to the cells in the plates and incubated for 2 h. The absorbance of the formazan produced by living cells was measured at 570 nm. The results were presented as the mean $\pm$ standard deviation (SD) of three independent experiments performed in triplicate. (ns: not significant; $p>0.05$, * ; $p<0.05$).

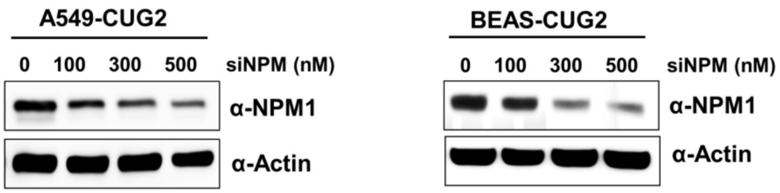

Supplementary Figure S2: Optimization of NPM1 siRNA concentration for NPM1 silence. A549-CUG2 and BEAS-CUG2 cells were treated with siNPM at the different doses (100, 300, and 500 nM) at 12 h post-inoculation. (siNPM1; NPM siRNA) At 48 h post-treatment, the cells were harvested and prepared for cell lysates. Down-regulation of NPM1 was detected by immunoblotting.

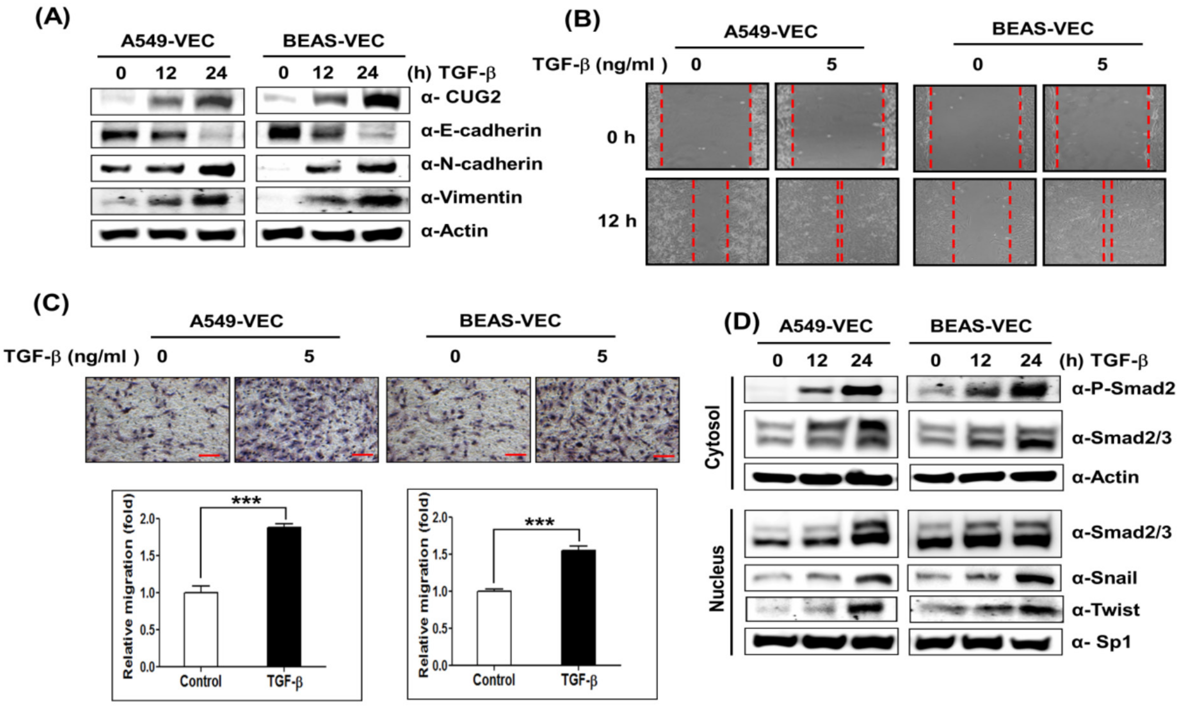

Supplementary Figure S3: TGF-β1 treatment induces EMT in A549-Vec and BEAS-Vec cells. A. Expression of CUG2, E-cadherin, N-cadherin, and vimentin in A549-Vec and BEAS-Vec cells was detected by immunoblotting at 12 h and 24 h post-treatment with TGF-β1 (5 ng/mL). **B.** Cell migration was measured in A549-Vec and BEAS-Vec cells at 24 h post-treatment with TGF-β1 (5 ng/mL) by a wound healing assay. The assays were repeated twice. **C.** An invasion assay was performed with A549-Vec and BEAS-Vec cells at 24 h post-treatment with TGF-β1 (5 ng/mL). The assays were repeated twice. Each assay was performed in triplicate and error bars indicate SD. (***; $p < 0.001$). **D.** A549-Vec and BEAS-Vec cells were treated with TGF-β1 (5 ng/mL) for 12 h and 24 h. The cells were fractionated into cytosolic and nuclear extracts. Expression of phospho-Smad2, Smad2/3, Snail, and Twist was detected by immunoblotting. Sp1 and actin were used loading controls for nuclear and cytosolic extracts, respectively.

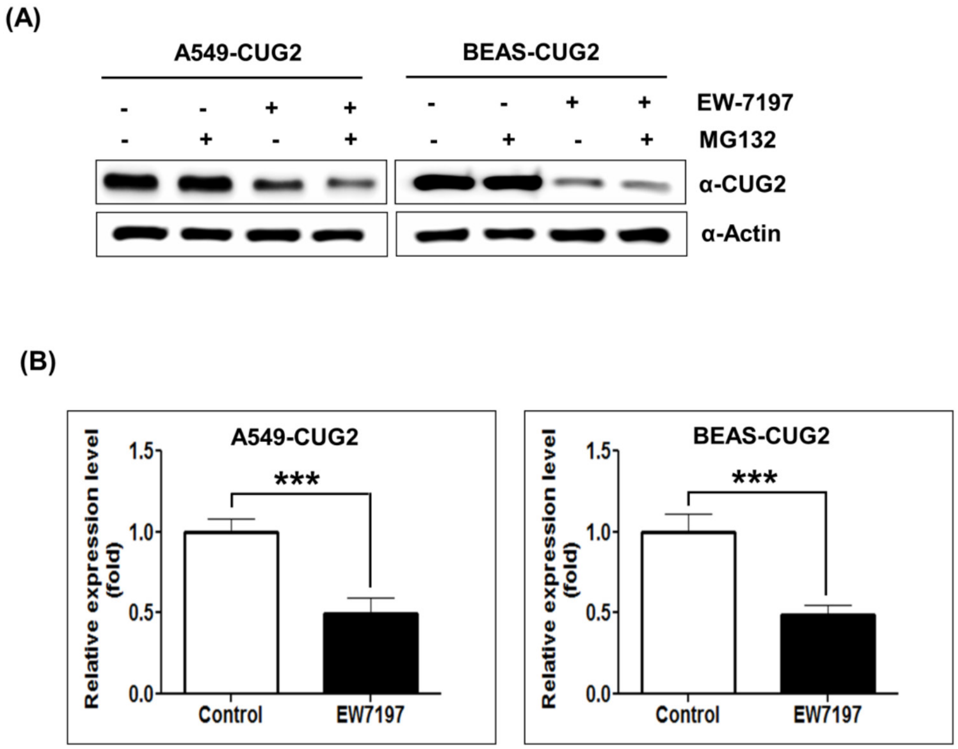

Supplementary Figure S4: EW-7197 treatment decreases CUG2 expression at the transcriptional level but not at the protein level. A. A549-CUG2 and BEAS-CUG2 cells were treated with EW-7197 (1.25 μM) and incubated for 48 h. At 8 h before harvest, MG132 (2 μM) was added in the cells and the cells were prepared for the detection of CUG2 protein levels by immunoblotting. **B.** A549-CUG2 and BEAS-CUG2 cells were treated with EW-7197 (1.25 μM) and at 24 h post-treatment, the cells were harvest for isolation of total RNAs. After synthesis of cDNAs, qRT-PCR was performed. The assay was repeated twice. Each assay was performed in triplicate. Error bars indicates SD. (***;$p<0.001$).

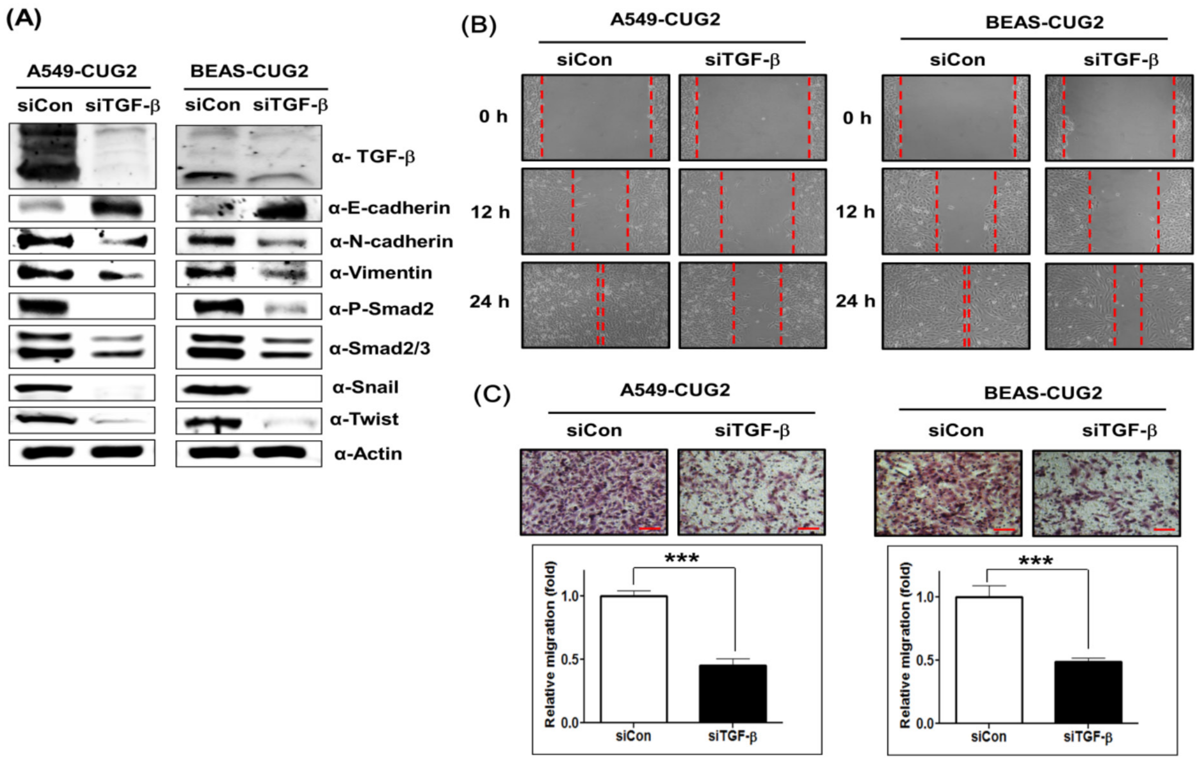

Supplementary Figure S5: TGF-β1 silence inhibits the CUG2-induced EMT. A. At 48 h post-treatment with TGF-β1 siRNA (500 nM), expression of TGF-β, E-cadherin, N-cadherin, vimentin, phospho-Smad2, Smad2/3, Snail, and Twist in A549-CUG2 and BEAS-CUG2 cells was detected by immunoblotting. (siCon; control siRNA, siTGF-β1; TGF-β1 siRNA) **B.** Cell migration was measured by a wound healing assay in A549- CUG2 and BEAS-CUG2 cells at 48 h post-treatment with TGF-β1 siRNA. The wound closure areas were monitored by phase-contrast microscopy at a magnification of 100×. The assays were repeated twice. **C.** An invasion assay was performed with A549-CUG2 and BEAS-CUG2 at 48 h post-treatment with TGF-β1 siRNA. Scale bar indicates 100 µm. The assays were repeated twice. Each assay was performed in triplicate and error bars indicate SD. (***; $p < 0.001$).

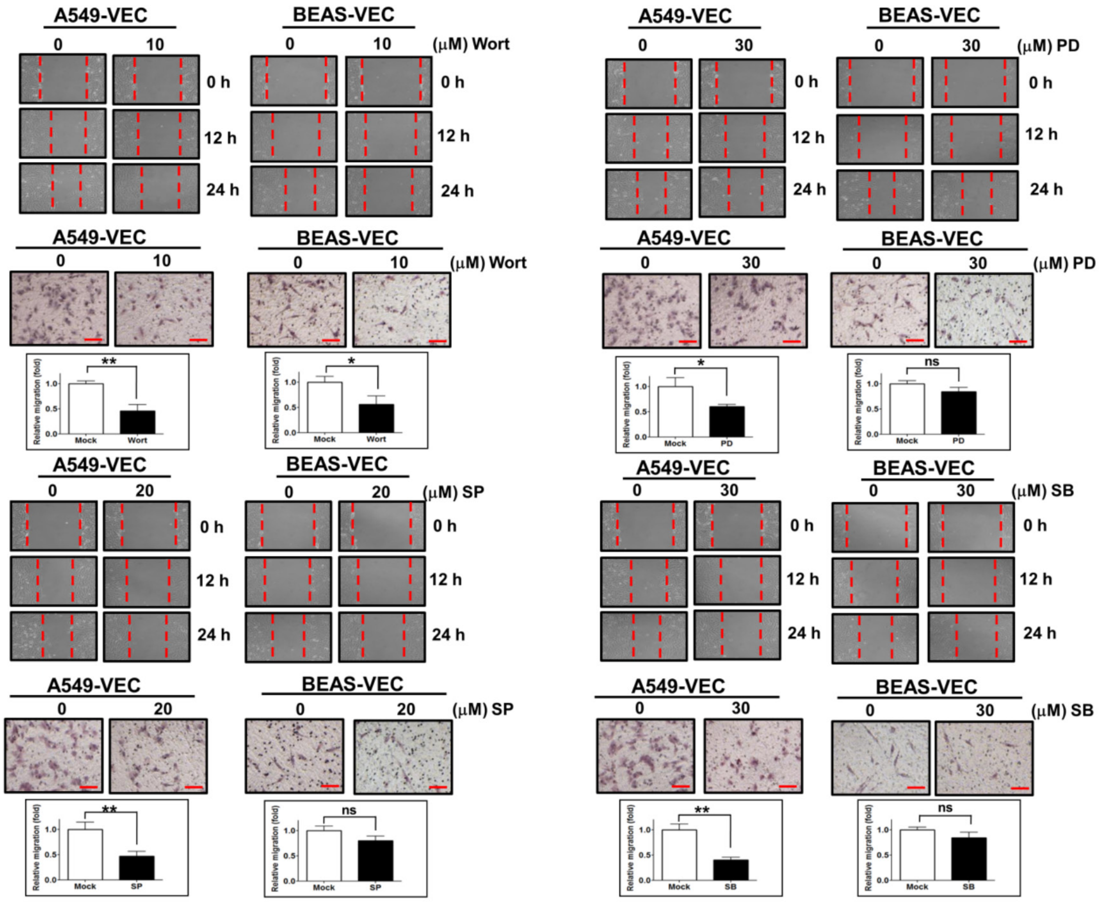

Supplementary Figure S6: Treatment with Akt or MAPK inhibitor marginally reduces EMT in A549-Vec and BEAS-Vec cells. A549-CUG2 and BEAS-CUG2 cells were treated with wortmannin (Wort), PD98059 (PD), SP600125 (SP), or SB203580 (SB). A wound healing assay was performed for cell migration. The wound closure areas were monitored by phase-contrast microscopy at a magnification of 100×. The assay was repeated twice. Cell invasion assay was performed using 48-well Boyden chambers. The chamber was assembled using polycarbonate filters coated with Matrigel. The assay was repeated twice. Each assay was performed in triplicate. Error bar indicates SD. (ns; $p>0.05$, *; $p< 0.05$, **; $p<0.01$).

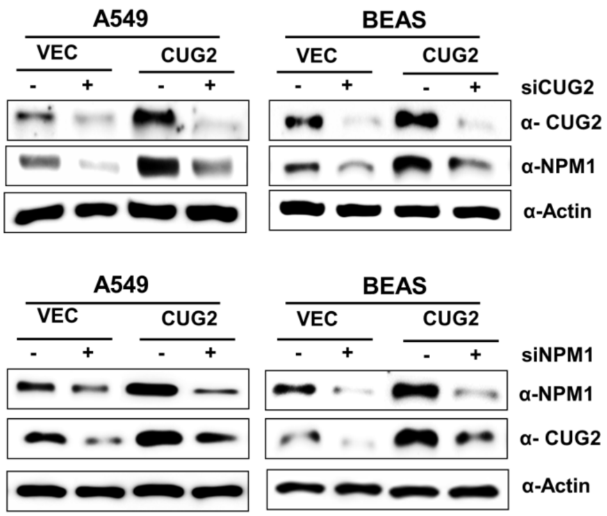

Supplementary Figure S7: Expression of both CUG2 and NPM1 shows interdependence. A549-CUG2 and BEAS-CUG2 cells were treated with CUG2 siRNA (500 nM) or NPM1 siRNA (500 nM). At 48 h post-treatment, the cells were harvested and CUG2 and NPM1 protein levels were detected by immunoblotting. (siCUG2; CUG2 siRNA, siNPM1; NPM1 siRNA).
